# Comparison of Illumina NovaSeq 6000, GeneMind SURFSeq 5000, Salus Evo, and MGI DNBSEQ-G400 for Ancient DNA Whole-Genome Sequencing

**DOI:** 10.3390/genes17080853

**Published:** 2026-07-24

**Authors:** Andrey D. Manakhov, Elizaveta V. Rozhdestvenskikh, Eleonora D. Aituganova, Aleksej N. Voroshilov, Natalia G. Svirkina, Svetlana S. Kunizheva, Tatiana V. Andreeva, Sergey N. Ostapenko, Vladimir D. Kuznetsov, Evgeny I. Rogaev

**Affiliations:** 1 Department of Genetics, Center for Genetics and Life Science, Sirius University of Science and Technology, Sirius Federal Territory 354340, Russia; rozenlizabet@gmail.com (E.V.R.); aytuganova2508@gmail.com (E.D.A.); voroshilov.aleksej@yandex.ru (A.N.V.); svirkina.natalia@mail.ru (N.G.S.); kunizheva@gmail.com (S.S.K.); antati12@gmail.com (T.V.A.); osn-23@mail.ru (S.N.O.); phanagor@mail.ru (V.D.K.); 2 Laboratory of Evolutionary Genomics, Department of Genomics and Human Genetics, Vavilov Institute of General Genetics of the Russian Academy of Sciences, Moscow 119991, Russia; 3 Institute of Archaeology Russian Academy of Sciences, Moscow 117292, Russia; 4 Centre of Genetics and Genetic Technologies, Faculty of Biology, Lomonosov Moscow State University, Moscow 119991, Russia; 5 The State Museum-Preserve «Phanagoria», Sennoy Village 353540, Russia; 6 Department of Psychiatry, UMass Chan Medical School, Shrewsbury, MA 01545, USA

**Keywords:** ancient DNA (aDNA), paleogenomics, next-generation sequencing (NGS), platform comparison, Illumina NovaSeq 6000, GeneMind SURFSeq 5000, Salus Evo, MGI DNBSEQ-G400, Phanagoria

## Abstract

**Background:** Ancient DNA (aDNA) research is one of the biological fields that has been transformed by the development of next-generation sequencing (NGS). To date, Illumina sequencing has dominated aDNA research. However, its high costs are driving the adoption of alternative sequencing platforms. Combining data generated on different platforms may introduce artifacts arising from platform-specific errors. This study systematically compared the performance of four sequencing platforms (Illumina NovaSeq 6000, GeneMind SURFSeq 5000, Salus Evo, and MGI DNBSEQ-G400) for whole-genome aDNA sequencing using the same set of six samples and libraries. **Methods:** Single-stranded libraries, prepared with and without enzymatic damage repair, were generated from six human aDNA specimens recovered from Phanagoria polis and sequenced on all four platforms. Data were subsampled to equal read counts per library, mapped to the human reference genome, and compared using standard NGS quality metrics. Population genetic analyses, including principal component analysis (PCA) and ADMIXTURE, were performed to assess potential platform-specific biases. **Results:** All platforms produced raw sequencing data of acceptable quality. Only minor differences were observed among platforms in standard NGS metrics. The MGI platform showed a shift toward longer sequenced fragment lengths compared with Illumina and the Illumina-like platforms. Post-mortem damage patterns, particularly C>T substitutions, were highly consistent across all platforms. PCA and ADMIXTURE analyses revealed no evidence of platform-specific bias: results from all platforms clustered tightly together, and platform choice had no significant effect on ancestry component estimates. **Conclusions:** Our findings demonstrate that the GeneMind, Salus, and MGI sequencing platforms are comparable to Illumina for paleogenomic research. Moreover, aDNA datasets generated on these platforms can be combined for downstream analyses without introducing detectable bias. However, the fragment-size shift observed on the MGI platform warrants caution and adaptation when working with highly degraded or low-endogenous-content samples.

## 1. Introduction

For the last 20 years, since the development and success of massively parallel or next-generation sequencing (NGS), there have been few areas of biology that have not adopted these techniques [1,2].

At the beginning of the NGS evolution, various methods and sequencing platforms were developed: Life Science/Roche 454, based on pyrosequencing; Solexa/Illumina, based on the reversible terminator method of sequencing combined with bridge amplification; SOLID and Complete Genomics, based on sequencing by ligation; and Ion Torrent, based on pH detection [1]. However, the Illumina platform emerged victorious and has maintained a dominant position in the sequencing market for an extended period, thanks to ongoing technological advancements and product commercialization [3].

As a result of improvements in data quality, read length, device throughput, and cost reduction, NGS applications are involved not only in fundamental but also in applied clinical research [4]. Moreover, novel research areas, such as ancient DNA (aDNA) investigations, have developed as a result of NGS technologies’ evolution [5].

Recently, despite the successes of the Illumina platform, alternative sequencing systems (GeneMind, SalusEvo, MGI, etc.) that are expected to combine cost-effectiveness, high accuracy, and flexibility have been developed in response to the growing demand for sequencing and the high cost of Illumina devices and reagents [3,4,6].

The previously mentioned area of ancient DNA studies is rapidly developing today. Modern studies require sequencing many decades and hundreds of samples [7,8,9], but due to low amounts of endogenous DNA and high levels of DNA degradation and damage, as well as contamination with DNA of soil microorganisms, the cost of whole-genome sequencing for a single aDNA sample is significantly higher than the cost of sequencing a single modern genome; therefore, reducing the cost is very important.

Only a few recent studies have used the GeneMind [10,11] or MGI [12,13,14,15] platforms for aDNA sequencing, but most of the ancient human genomes available in databases were obtained using Illumina sequencing technology. For instance, we observe only a single study [16] with samples sequenced on a platform other than Illumina in the AADR v62.0 database [17], which has over 10,000 human aDNA samples from publications spanning 2010 to 2024.

Considering that the majority of aDNA research uses pseudo-haploid genotype variant calling based on a single read, combining data from several sequencing platforms that each have individual sequencing errors may introduce artifacts during analysis.

In this study, we investigated the results of whole-genome sequencing of a set of six aDNA samples and libraries executed via four different platforms: Illumina, GeneMind, SalusEvo, and MGI.

## 2. Materials and Methods

Paleoanthropological material from the collections of the State Museum-Preserve “Phanagoria” and the Archaeological Institute RAS was used. The remains originate from burials in the southern and eastern necropolises, as well as from the cultural layer of the Phanagoria polis. Archaeological examination by the Phanagoria expedition of the Archaeological Institute RAS, as well as radiocarbon dating performed in the AMS Golden Valley laboratory (Novosibirsk, Russia), indicates that the remains belong to the Phanagoria polis populations from the Classical to Khazar periods of their history (Table 1).

Genomic DNA was extracted from fragments weighing 100–200 mg in a specialized lab for work with aDNA at Sirius University, according to a previously published method [18]. Fragmented genomic libraries were prepared according to a single-stranded DNA-based protocol [19]. From each aDNA sample, two libraries, with and without repair using PreCR Repair Mix (NEB, Ipswich, MA, USA) [20], were prepared. Sequencing was performed on the Illumina NovaSeq 6000 instrument at Sirius University (Sirius Federal Territory, Russia) in the single-read 56 and 121 bp mode; on the GeneMind SURFSeq 5000 instrument at Genomed Company (Moscow, Russia), in the paired-end 120 bp mode; and on the Salus Evo instrument at R-gene Company (Moscow, Russia), in the paired-end 120 bp mode. For sequencing on the MGI platform, we performed a conversion of the libraries into circular single-strand libraries using the MGI Easy Universal Library Conversion Kit (App-A, Shenzhen, China) according to the manufacturer’s protocol. Sequencing was performed using the MGI DNBSEQ-G400 instrument at the Petrovskiy Russian Research Center of Surgery (Moscow, Russia), in the single-read 56 bp mode.

To perform an accurate comparison of the sequencing results obtained with four different platforms, we used only the first reads cropped to the same length (56 bp) with a custom Python v3.10.6 script. Additionally, we performed random subsampling with SeqKit v2.4 [21] to get nearly the same number of reads per sequenced library.

Trimming of the low-quality bases, Ns, and adapter sequences was performed with AdapterRemoval v2.3.1 software [22]; reads shorter than 25 nucleotides were eliminated from further analysis. The resulting reads were mapped against both the human reference genome (GRCh37) and the revised Cambridge Reference Sequence (rCRS, NC_012920) with BWA v0.7.17 software [23] using the parameters (-n 0.01 -o 2 -l 1024) adopted for aDNA sequencing results [24]. Duplicates were marked by the MarkDuplicates function from the Picard toolkit v2.25.4 [25].

Determination of the post-mortem pattern specific to the aDNA and rescaling of the base quality scores were performed separately for each library with mapDamage2 v2.2.1 software [26]. Genetic sex of individuals was determined based on reads with mapping quality scores greater than 30 using the karyo_RxRy v1.0.1 software [27]. We used the Schmutzi v1.5.5.5 [28] and ContamMix v1.0.11 [29] software to estimate contamination based on mtDNA. Additionally, the ANGSD v0.937 software was used to estimate contamination on the X chromosome for a male individual [30].

A pseudo-haploid dataset was generated with the pileupCaller function from the SequenceTools v1.4 software [31] by randomly (randomHaploid parameter) selecting one read per SNP of the 1240K SNP panel presented in AADR v.62.0. We used subsampling to achieve identical coverage results of repaired libraries’ sequencing. Reads with MQ > 30 and BQ > 30 were used for pseudo-haploid variant calling.

Population analysis was performed using principal component analysis (PCA) using the smartpca function from the EIGENSOFT v7.2.1 software package [32]. For this purpose, the genotype profiles of the studied samples from Phanagoria were projected onto the genetic variability of present-day Eurasian, European, and Caucasian populations from the Human Origin (HO) panel [33].

To evaluate the ancestry composition, we performed an unsupervised ADMIXTURE [34] analysis using a total of 4666 samples (including 6 of our samples sequenced across four different panels; 4417 ancient samples from the AADR v62 database (dating from 5700 to 600 years ago); and 80 ancient and 169 modern samples from the AADR v62 database, in which ancestral components generally accepted in palaeogenetic research are maximised), genotyped with 600K SNPs from the HO panel. For every kinship pair or family group, we selected the single individual with the greatest number of SNPs. We used PLINK v2.00 [35] to exclude variants present in only one individual and performed pruning for linkage disequilibrium (–indeppairwise 200 5 0.5). ADMIXTURE v1.3 was run with default parameters, and the number of ancestral populations ranged between K = 1 and K = 12.

## 3. Results

This study, to the best of our knowledge, is the first to systematically compare the performance of the Illumina NovaSeq 6000, GeneMind SURFSeq 5000, Salus Evo, and MGI DNBSEQ-G400 platforms (Table 2) in aDNA whole-genome sequencing research.

The Illumina NovaSeq 6000 uses a sequencing workflow, which is based on in situ amplification of single-stranded DNA templates in nanowells on the flow cell surface and sequencing by synthesis (SBS). During each sequencing cycle, a single fluorescently labeled deoxynucleotide triphosphate (dNTP) is added to the nucleic acid chains. The fluorescent dye acts as a reversible terminator and, before its removal, prevents the addition of subsequent dNTPs to the growing chain. In each sequencing cycle, fluorescent emissions from each cluster are recorded and used for base-calling [36].

The GeneMind SURFSeq 5000 sequencer also employs surface-based amplification and SBS techniques, similar to those used by Illumina. It is also based on reversible termination approaches and incorporates proprietary features intended to enhance efficiency [6].

The Salus Evo, the third Illumina-type sequencing platform used in our study, also utilizes bridge PCR technology and the reversible terminator method for SBS.

The MGI DNBSEQ-G400 platform employs the combinatorial Probe–Anchor Synthesis (cPAS) method, which relies on DNA nanoballs (DNBs). Single-stranded DNA templates are circularized and undergo rolling circle amplification to produce DNBs, which are then immobilized on the patterned array flow cell surface. During each sequencing cycle, a fluorescently labeled dNTP probe is incorporated into the DNA strand by DNA polymerase. After imaging and decoding of the fluorescent signal, regeneration reagents remove the fluorescent dye and prepare the DNBs for the next sequencing cycle.

We performed parallel sequencing of the same libraries (in the case of MGI, converted from Illumina to MGI format) prepared according to a single-stranded DNA-based protocol, which is more efficient in cases when small quantities of highly degraded aDNA are present [37]. Comprehensive sequencing results and key statistics for all samples and libraries, subsampled to the same total number and length of reads, are summarised in Table S1.

### 3.1. Comparing Base NGS Data Parameters

**Figure 1:**
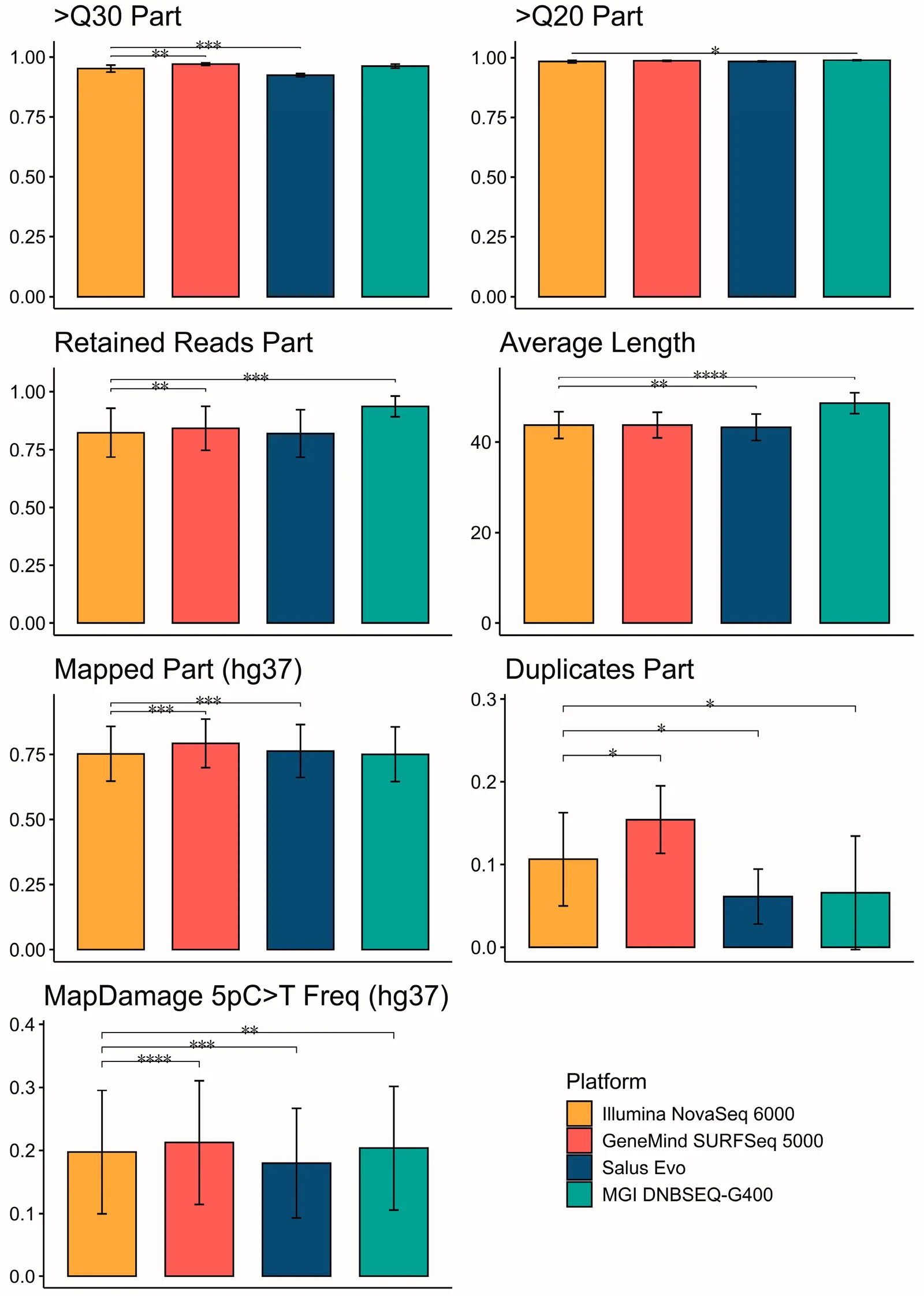
Statistics of base NGS data parameters for all platforms. Bars indicate the mean value, and whiskers indicate the standard deviation across 12 values per platform. Comparison with Illumina NovaSeq 6000 data was performed using a paired *t*-test, corrected for multiple comparisons using the Holm method. Significance level of *p*.adj: * <0.05, ** <0.01, *** <0.001, **** <0.0001.

The sequence quality of the raw data from all platforms was acceptable for further analysis; however, for the “>Q30 (%)” metric, GeneMind (96.9 ± 0.53%) demonstrates significantly higher values than Illumina (95.1 ± 1.45%), while Salus Evo (92.3 ± 0.68%) shows significantly lower values (Figure 1). All platforms demonstrated similar values for the “>Q20 (%)” metric (≈98%); the slight preponderance was observed only in the MGI DNBSEQ-G400 data, compared with Illumina.

The average length of retained reads and the proportion of reads retained following adaptor removal were comparable between the Illumina and Illumina-like (GeneMind and Salus Evo) platforms. When compared to NovaSeq 6000 (0.823 ± 0.106 and 43.7 ± 2.94 bp), SURFSeq 5000 shows a modest increase in the retained reads section (0.842 ± 0.095), while Salus Evo shows a minor drop in the average length of retained reads (43.2 ± 2.91 bp). This difference appears to be the consequence of the above-mentioned variation in sequence quality.

For the MGI platform, we observe a significant increase in both parameters, the proportion of reads retained (0.936 ± 0.0446) and their average length (48.5 ± 2.29 bp). We checked the distribution of read length after adaptor removal and observed that for MGI it was shifted towards longer fragments compared with Illumina and Illumina-like platforms (Figure S1). This appears to be a consequence of the library conversion process, which is necessary to turn linear Illumina library fragments into circular fragments for further rolling circle amplification and DNA nanoball formation. Previously, it was noted that the circularization step may result in fragment size selection or reduced ligation efficiency for short DNA fragment sizes [38].

Analysis of data mapped against the human reference genome revealed that Illumina-like platforms demonstrated a significant increase in the “Endogenous DNA rate” metric compared to Illumina (0.752 ± 0.105), with Salus Evo (0.762 ± 0.102) slightly elevated, and GeneMind (0.792 ± 0.093) showing the greatest increase. No significant differences were observed for the MGI platform.

Regarding the “Duplicate rate” parameter, we observed significant differences from Illumina (0.106 ± 0.056) across all platforms. For the Salus Evo platform, the lowest portion of mapped reads was marked as duplicates (0.061 ± 0.033). We associate it with different flow cell organization types (patterned for Illumina NovaSeq and random for Salus Evo). The 1500M Salus Evo flow cell used in this study is a random type; however, a higher-throughput 3000M flow cell is patterned. It is known that Illumina-patterned flow cells demonstrate a higher duplication rate compared with random ones [39,40]. During library conversion to MGI type, Illumina libraries were additionally amplified with 10–15 PCR cycles; however, the proportion of duplicates (0.066 ± 0.069) was lower than on the Illumina platform and did not differ significantly from that on the Salus Evo platform (*p*-value = 0.701). For the GeneMind SURFSeq 5000 platform, we observed an increase in the “Duplicate rate” parameter value (0.154 ± 0.041) compared with Illumina NovaSeq (both platforms have patterned flow cells).

Detection of post-mortem DNA damage patterns is one of the key steps in aDNA sequencing results analysis. On the one hand, this damage limits our ability to perform experimental and bioinformatic work with aDNA; on the other hand, signs of present deaminated forms of cytosines (uracils) in reads are a powerful approach to authenticate aDNA sequences [26]. We observed significant but slight differences in the frequency of C>T substitutions at the 5′ end of sequenced fragments across platforms (Figure 1, Table S1). However, the profiles of C>T substitutions over 25 bp at the 5′ end of fragments were identical across all platforms (Figure S2).

### 3.2. Population Genetic Analyses

To further explore whether there are platform-specific biases in population genetic analyses of aDNA sequencing results, we performed the two most common analyses in paleogenomics: principal component analysis (PCA) and ADMIXTURE. For this analysis, we used only the sequencing results from the repaired libraries, mapped against the human reference genome, and additionally subsampled (per sample) to the same coverage (Table S2). No significant differences in the number of genotyped SNPs were observed between platforms.

**Figure 2:**
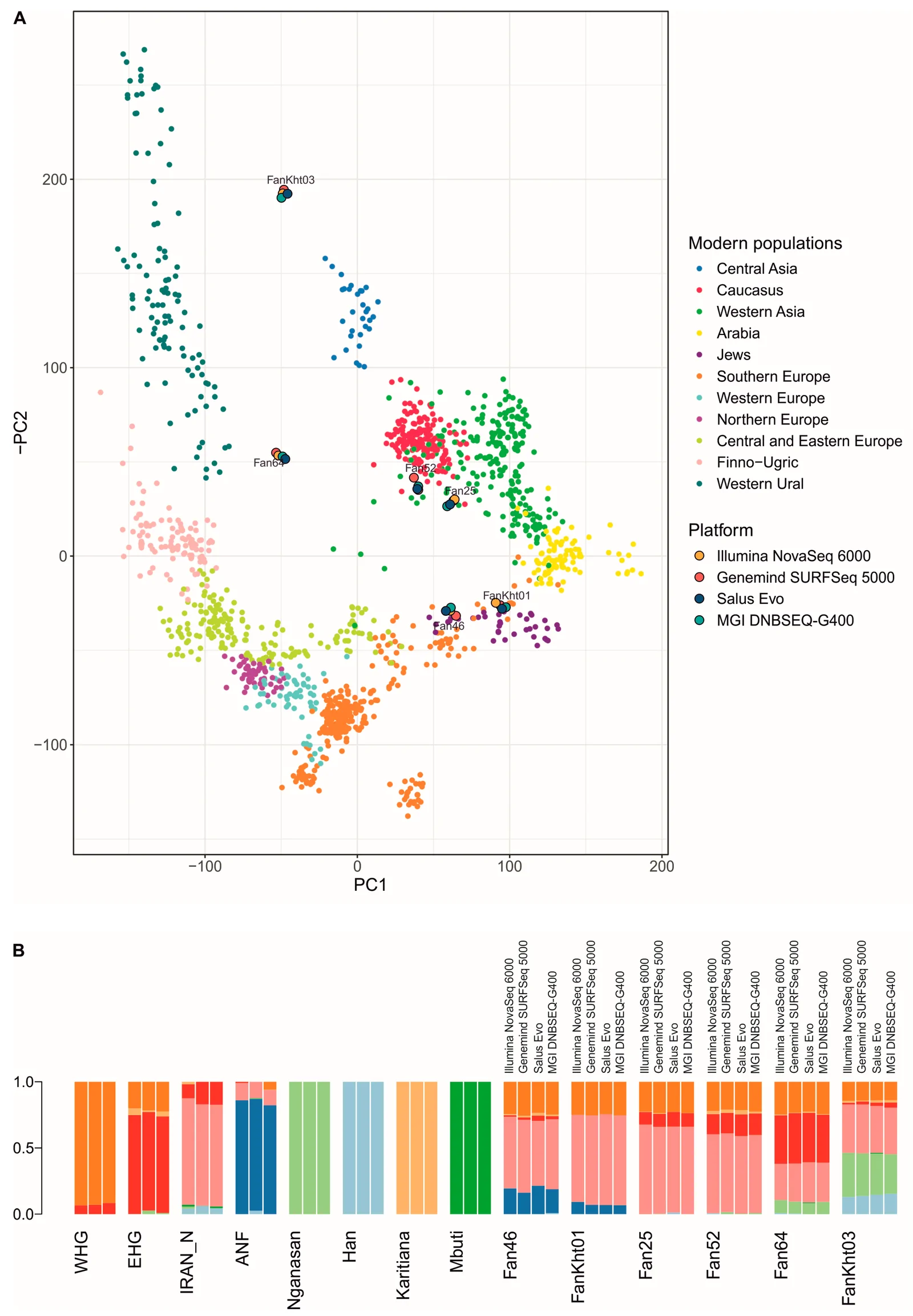
PCA and ADMIXTURE analysis. (**A**) Projection of ancient samples (circles filled by platform) onto the first two principal component variations (PC1 and PC2) of present-day Western Eurasian and Caucasus individuals (points coloured by geographical groups); (**B**) ADMIXTURE profiles (K = 8) for studied individuals and basic populations. WHG—Western hunter-gatherers; EHG—Eastern hunter-gatherers; IRAN_N—Iranian Neolithic; ANF—Anatolian Neolithic Farmers.

According to PCA, the same samples from all platforms were projected closely together; in most cases, points overlapped (Figure 2A and Figure S3). ADMIXTURE results were consistent with the PCA clustering results and indicated that the same samples from all platforms had highly similar profiles (Figure 2B). A one-way ANOVA analysis showed no significant effect of the sequencing platform on the composition of ancestry components in ADMIXTURE results.

## 4. Discussion

Previously, multiple studies demonstrated the comparability of data obtained from the Illumina, GeneMind, and MGI platforms for genome [41], exome [3], transcriptome [4,42], single-cell [6], and target [38] studies. However, comparative studies of various platforms in the field of aDNA sequencing are not well represented. We observe only two studies comparing Illumina HiSeq 2500 with BGISEQ-500 [43] and Illumina HiSeq X with MGI DNBSEQ-G400 [44]. Both of them demonstrated that MGI can be used as a potential alternative to Illumina for paleogenomic sequencing. It should be noted that both Illumina HiSeq machines were replaced several years ago with more high-throughput and cost-effective devices, such as the NovaSeq 6000 and NovaSeq X.

In this study, for the first time, we use four different sequencing platforms for whole-genome aDNA sequencing. Comparing sequencing results obtained with the GeneMind SURFSeq 5000, Salus Evo, and MGI DNBSEQ-G400 platforms with the Illumina NovaSeq 6000 data, we detected significant differences in some parameters (base quality, mapping, and duplicate rates) of high-throughput sequencing datasets; however, the magnitude of these differences seems to be slight.

On the MGI sequencing platform, we identified significant differences in the length of sequenced DNA fragments that seem to be the result of fragment size selection or low ligation efficiency of short DNA fragments during the circularization step. However, in our study, based on relatively good aDNA samples with high levels of human endogenous DNA (>60%), we did not observe a significant impact of this feature. However, it requires further investigation and may be crucial for more degraded and/or contaminated samples (e.g., soil microorganism DNA).

Regarding the population genetics analysis, we did not observe any platform-specific bias; the results of PCA and ADMIXTURE analyses based on data from different sequencing technologies demonstrated a high level of convergence.

As was mentioned above, our study was based on samples with high levels of human endogenous DNA. Data obtained from this set of samples suggest that GeneMind, Salus, and MGI sequencing platforms can be used as cost-effective alternatives to Illumina in paleogenomics research. However, further investigations with numerous, degraded, and more varied aDNA samples should be performed to reflect all conditions encountered in ancient samples.

## Figures and Tables

**Table 1:** Archaeological data and results of the radiocarbon dating regarding the analyzed samples.

Sample ID	Burial	Date ^1^	Sex ^2^	Age, Years	Radiocarbon Dating		
					Laboratory Number	Radiocarbon Determination	Calibrated Data INCAL20, 95.4%
Fan25	Eastern necropolis, 256/2013 1	Hellenistic period, 2 c. BCE	M ^3^	10	GV-6662	2093 ± 23 BP	172–45 calBCE
Fan46	Southern necropolis, 288/2012 1	Classical period, first quarter 4 c. BCE	F	≈50	GV-5850	2500 ± 36 BP	784–483 calBCE
Fan52	Eastern necropolis, 194/2013	Roman period	M	40–49	GV-6663	2276 ± 24 BP	400–209 calBCE
Fan64	Eastern necropolis, 226/2014	Roman period	M ^3^	10–11	GV-6025	2069 ± 36 BP	176 calBCE–24 calCE
FanKht01	Upper Town, Object 749/2016	2 c. CE	M	≈50	GV-6026	2038 ± 35 BP	154 calBCE–65 calCE
FanKht03	Lower Town, Object 10/2016, spit 15	Khazar time, 7–9 c. CE	–	6–7	GV-6028	1288 ± 35 BP	658–821 calCE

^1^ Based on archaeological data; ^2^ based on anthropological data; ^3^ uncertain sex determination.

**Table 2:** Comparison of key parameters of the sequencing platforms and reagent kits used in this study.

Platform	Reagent Kit	Flow Cell Type	Ampification Type	Sequencing Chemistry Type	Read Number (M) Yield	
					Expected	Observed
Illumina NovaSeq 6000	SP Reagent Kit v1.5 (100 cycles)	Patterned	Bridge	2-colour	800	940 ± 70 ^1^
	S4 Reagent Kit v1.5 (35 cycles)				10,000	12,659 ± 743 ^2^
GeneMind SURFSeq 5000	Sequencing Kit V2.0 (FCP 300 cycles)	Patterned	Bridge	4-colour	3600	4225
Salus Evo	PRH-PE-150-1500M	Random	Bridge	4-colour	1500	1876
MGI DNBSEQ-G400	DNBSEQ-G400RC FCL SE100	Patterned	Rolling circle	4-colour	1500	2190

^1^ Based on 11 runs of data; ^2^ based on 34 runs of data.

## Data Availability

The datasets generated during the current study were deposited into the NCBI SRA database and can be accessed with the BioProject accession number PRJNA1480240 (https://www.ncbi.nlm.nih.gov/bioproject/PRJNA1480240 (accessed on 21 July 2026)).

## Supplementary Materials

The following supporting information can be downloaded at https://www.mdpi.com/article/10.3390/genes17080853/s1, Figure S1: The distribution of the read length after adaptor removal. Figure S2: The profile of the C>T transitions specific to ancient DNA for reads mapped to the hg37 reference genome, calculated with MapDamage2. The X axis denotes the nucleotide position from the 5′ end of the DNA fragments. Figure S3: Projection of ancient samples (circles filled by platform) onto the first two principal component variations (PC1 and PC2) of present-day Eurasians (points coloured by geographical groups). Table S1: Summary statistics of key parameters of whole-genome sequencing and contamination level estimation, normalized by read number and length for each library dataset. Table S2: Genome coverage and counts of genotyped SNPs after depth normalization of whole-genome sequencing based on repaired aDNA.
