# Supplementary material for: Comparison of Illumina NovaSeq 6000, GeneMind SURFSeq 5000, Salus Evo, and MGI DNBSEQ-G400 for Ancient DNA Whole-Genome Sequencing
Source: Genes (Basel). 2026 Jul 24;17(8):853. doi: 10.3390/genes17080853 (PMC13512658; doi:10.3390/genes17080853)
Supplement: Supplementary file 1 [file genes-17-00853-s001.zip › Figure S1.pdf]

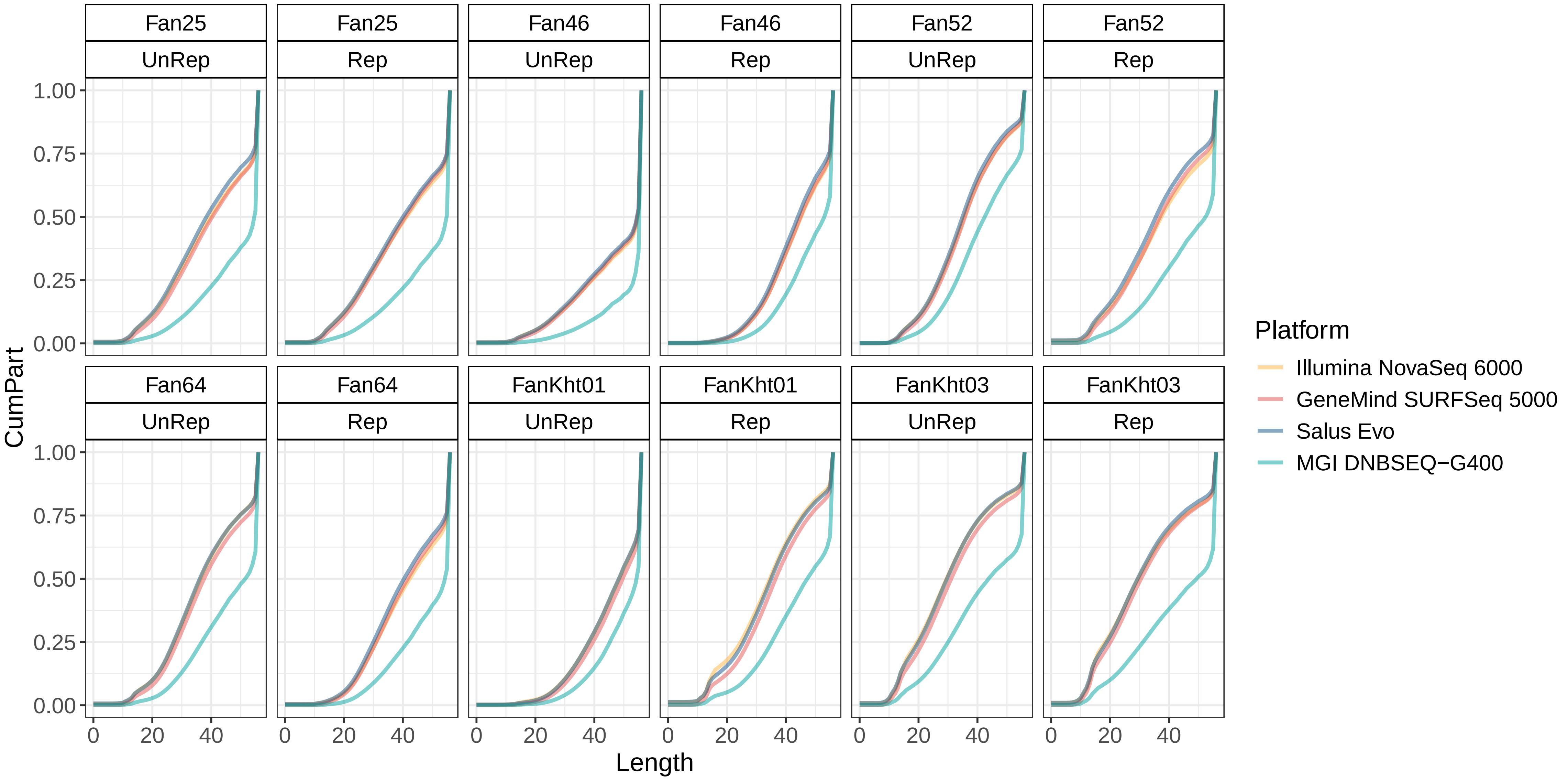

Fan25
UnRep
Fan25
Rep
Fan46
UnRep
Fan46
Rep
Fan52
UnRep
Fan52
Rep
Fan64
UnRep
Fan64
Rep
FanKht01
UnRep
FanKht01
Rep
FanKht03
UnRep
FanKht03
Rep
CumPart
1.00
0.75
0.50
0.25
0.00
0
20
40
Length
Platform
Illumina NovaSeq 6000
GeneMind SURFSeq 5000
Salus Evo
MGI DNBSEQ−G400
