# Supplementary material for: Comparison of Illumina NovaSeq 6000, GeneMind SURFSeq 5000, Salus Evo, and MGI DNBSEQ-G400 for Ancient DNA Whole-Genome Sequencing
Source: Genes (Basel). 2026 Jul 24;17(8):853. doi: 10.3390/genes17080853 (PMC13512658; doi:10.3390/genes17080853)
Supplement: Supplementary file 1 [file genes-17-00853-s001.zip › Figure S2.pdf]

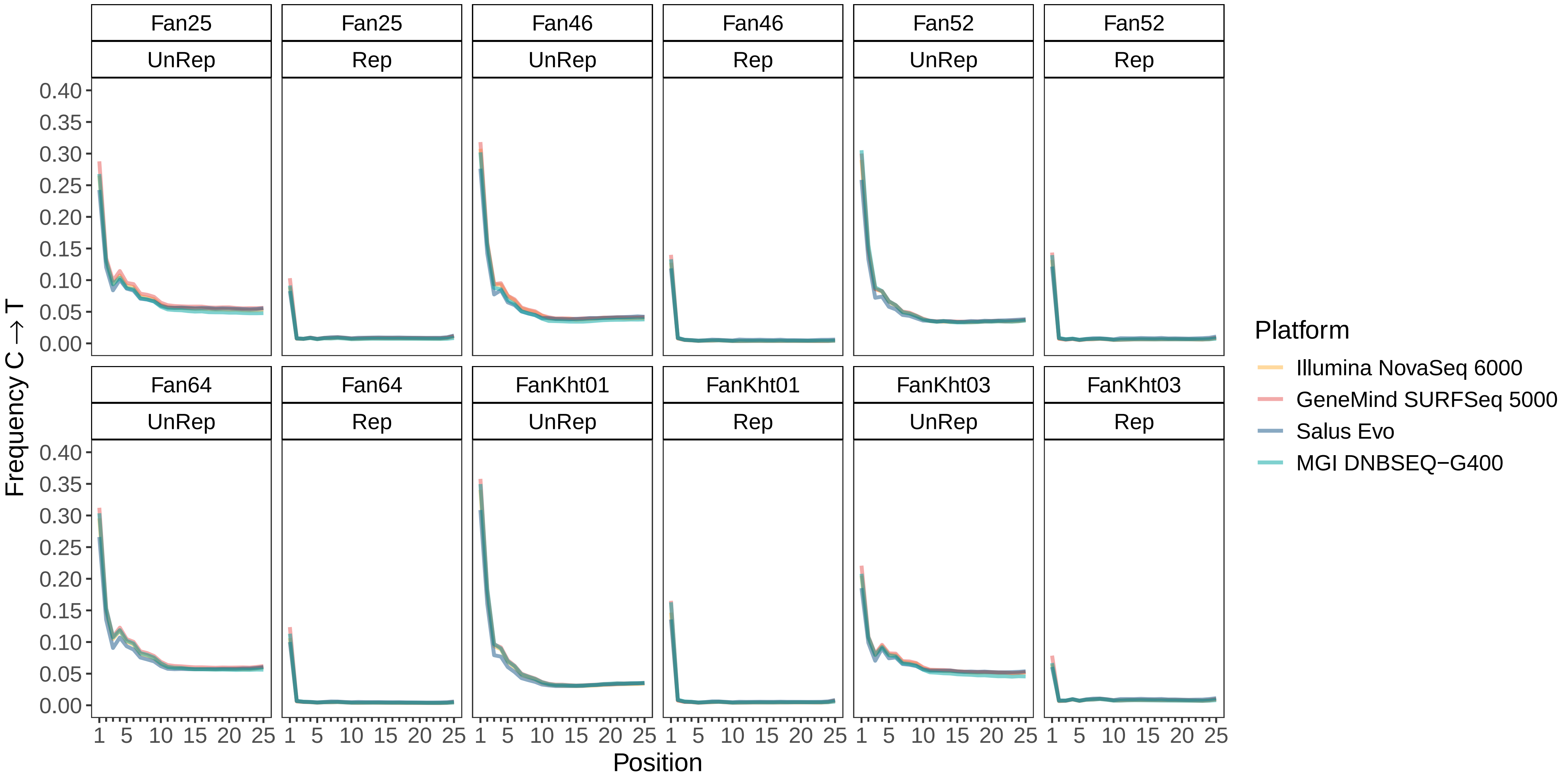
Fan25 UnRep
Fan25 Rep
Fan46 UnRep
Fan46 Rep
Fan52 UnRep
Fan52 Rep
Fan64 UnRep
Fan64 Rep
FanKht01 UnRep
FanKht01 Rep
FanKht03 UnRep
FanKht03 Rep
Frequency C → T
0.00 0.05 0.10 0.15 0.20 0.25 0.30 0.35 0.40
1 5 10 15 20 25
Position
Platform
Illumina NovaSeq 6000
GeneMind SURFSeq 5000
Salus Evo
MGI DNBSEQ−G400
