# Supplementary material for: Comparison of Illumina NovaSeq 6000, GeneMind SURFSeq 5000, Salus Evo, and MGI DNBSEQ-G400 for Ancient DNA Whole-Genome Sequencing
Source: Genes (Basel). 2026 Jul 24;17(8):853. doi: 10.3390/genes17080853 (PMC13512658; doi:10.3390/genes17080853)
Supplement: Supplementary file 1 [file genes-17-00853-s001.zip › Figure S3.pdf]

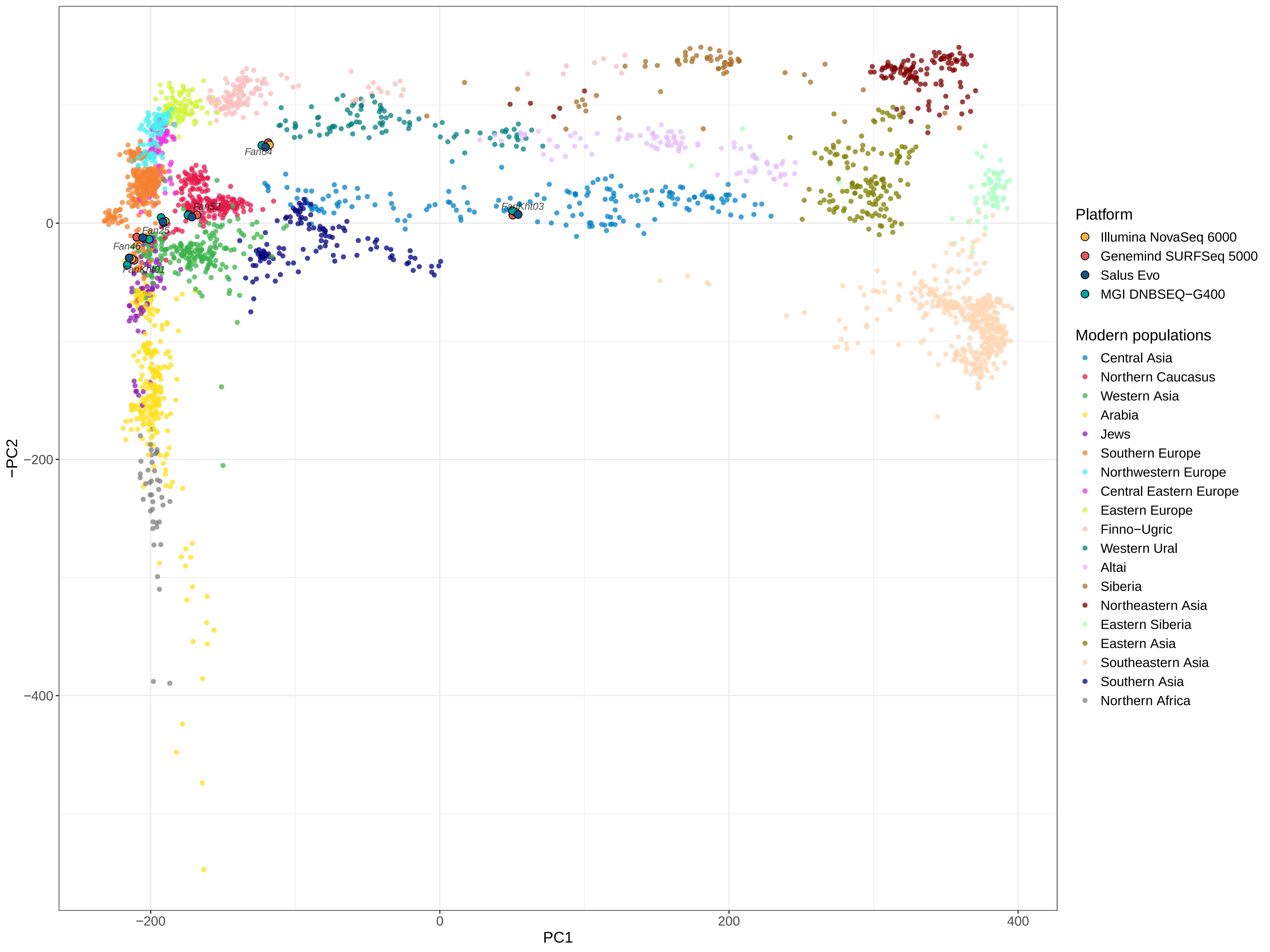

Fan04
Fan52
Fan25
Fan46
FanKht01
FanKht03
0
−200
−400
−PC2
−200
0
200
400
PC1
Platform
Illumina NovaSeq 6000
Genemind SURFSeq 5000
Salus Evo
MGI DNBSEQ−G400
Modern populations
Central Asia
Northern Caucasus
Western Asia
Arabia
Jews
Southern Europe
Northwestern Europe
Central Eastern Europe
Eastern Europe
Finno−Ugric
Western Ural
Altai
Siberia
Northeastern Asia
Eastern Siberia
Eastern Asia
Southeastern Asia
Southern Asia
Northern Africa
